# Hybrid Two-Scale Fabrication of Sub-Millimetric Capillary Grippers

**DOI:** 10.3390/mi10040224

**Published:** 2019-03-29

**Authors:** Sam Dehaeck, Marco Cavaiani, Adam Chafai, Youness Tourtit, Youen Vitry, Pierre Lambert

**Affiliations:** 1 TIPs Department, Université libre de Bruxelles (ULB), 1050 Brussels, Belgium; sam.dehaeck@gmail.com (S.D.); adam.chafai@ulb.be (A.C.); ytourtit@ulb.ac.be (Y.T.); yvitry@ulb.ac.be (Y.V.); 2 Politecnico di Milano, 20133 Milano, Italy; marco.cavaiani@outlook.com

**Keywords:** capillary gripper, pick and place, micromanufacturing, two-photon polymerization, stereolithography, polydimethylsiloxane (PDMS) replication

## Abstract

Capillary gripping is a pick-and-place technique that is particularly well-suited for handling sub-millimetric components. Nevertheless, integrating a fluid supply and release mechanism becomes increasingly difficult to manufacture for these scales. In the present contribution, two hybrid manufacturing procedures are introduced in which the creation of the smallest features is decoupled from the macro-scale components. In the first procedure, small scale features are printed directly (by two-photon polymerisation) on top of a 3D-printed device (through stereolithography). In the second approach, directional ultraviolet (UV)-illumination and an adapted design allowed for successful (polydimethylsiloxane, PDMS) moulding of the microscopic gripper head on top of a metal substrate. Importantly, a fully functional microchannel is present in both cases through which liquid to grip the components can be supplied and retracted. This capability of removing the liquid combined with an asymmetric pillar design allows for a passive release mechanism with a placement precision on the order of 3% of the component size.

## 1. Introduction

Micromanipulation can be based on a wide variety of physical principles (dielectrophoresis, magnetic, optical tweezers, mechanical tweezers, hydrodynamic flows … [1]). However, industrial picking-and-placing of sub-millimetric components such as SMD (surface mounted device) components is dominated by vacuum gripping. Very efficient even for components as small as 200 μm, this technique reaches its limits for smaller components because of scaling laws (adherence force scales as the square of the size [2]). Another challenge for such grippers in handling micro-components comes from the need to keep the collision force minimal. As discussed in literature [3], specialised solutions such as air bearings for the gripper head are necessary to increase the picking frequency. This added complexity is not without its cost.

An emerging alternative is the capillary picking paradigm [4], relying on capillary forces generated by the liquid meniscus between the component and the picking tool. Here, the adhering force is linear with the component size, which scales better than vacuum gripping for smaller components. This liquid layer also acts as a cushion so the component remains scratch free and surface irregularities do not impact the picking force substantially. The main design choices that need to be addressed for such a gripper are the liquid handling strategy (through a channel, dipping or evaporation/condensation) and the releasing mechanism (active/passive) [5,6,7]. By externalising these two mechanisms, the gripper head can be made quite simple (e.g., a simple polysterene sphere glued to a golden rod [7]) at the expense of a more elaborate control of the exterior conditions (temperature control of the gripper and the substrate [7]). In the present contribution, we will demonstrate a gripper design where most of the complexity is integrated in the gripper head so as to minimise the demands on the ambient control. As a result, the manufacturing of such a gripper presents some issues, mainly due to the multiplicity of scales present: the gripper surface will be of a size similar to the component and this will need to be connected to cm-sized components (mechanical stage and liquid supply) and finally, a releasing mechanism or surface texturation might be present which will be an order of magnitude smaller than the component.

One manufacturing technique capable of creating complicated 3D shapes with micro-meter resolution is two-photon polymerisation [8], as was demonstrated by e.g., the manufacturing of a vision-based force-sensor [9]. The challenge here is more in reaching the centimetric scale with this kind of machine. Although ‘meso-scale’ printing with this technique has been improved with respect to manufacturing time [10] and the removal of defects [11], it remains a point-wise polymerisation technique with a micron-sized point. Therefore, polymerising a centimetric object will inherently require an excessive amount of time.

To overcome this, two different hybrid two-scale manufacturing approaches are suggested. In this way, the gripper itself is created with two-photon polymerisation and the larger scales are handled by other manufacturing techniques. While this is somewhat similar to the work of [12], we will here focus more on the assembling of different manufacturing techniques. In Section 3, we will combine two different 3D printers and in Section 4 a replication strategy for fast serial production of grippers is shown. After the description of the two manufacturing techniques, the correct functioning of the gripper is shown and a novel passive releasing mechanism is explained. Note that the first assembly is based on the work described in [13].

## 2. Design Overview

**Figure 1:**
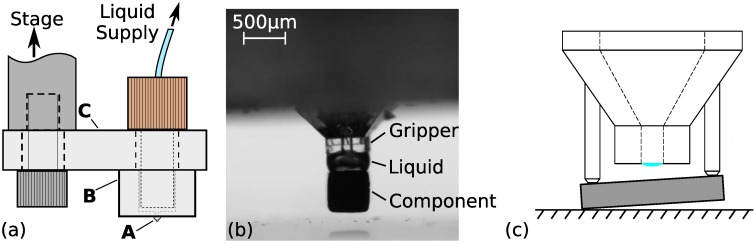
(**a**) Global overview of design (**b**) Example of capillary gripping [13] (**c**) Sketch of releasing mechanism based on unequal pillars design.

Before introducing the different hybrid manufacturing techniques that were used for the construction of our capillary grippers, we will first present their global design and working principle. In Figure 1a, a macroscopic sketch is given of the overall design. The part that is performing the capillary gripping is the sub-millimetric component indicated by the letter A in the figure. This small size is determined by the target that we want to pick up; in this case a 1005 SMD capacitor, which has a 1.0 × 0.5 (mm) surface. In Figure 1b, we can see a simplified version of the gripper in action. A liquid bridge between the two flat surfaces provides the force to lift the component. Releasing of the target should occur upon retraction of the liquid. However, when the target thus comes in contact with the gripper surface, some remaining liquid in the gap will typically prevent the release. Therefore, the unequal side-pillar design shown in Figure 1c is proposed as a passive release mechanism. As the target has a rectangular shape of 1.0 × 0.5 (mm) and our gripping surface is only 0.5 × 0.5 (mm), the target will have a large overhang in one of the directions. Now, upon withdrawing the liquid into the channel, this overhanging part will be blocked at the location of the two unequal-sized pillars and this leads to a slant of the component. A gentle downward movement of the gripper, will lead to a detaching moment when the corner of the target touches the substrate. This results in the controlled release of the component. More details on the functioning principle and a demonstration will be given in Section 5.

Although the design would be simplified by the absence of the liquid supply line, it forms an integral part of the operation here. As such, the gripper head needs to be connected to a (larger-scale) liquid injection system. This part, which we will call the tip-holder, is denoted by B in Figure 1a. Finally, part C is necessary to connect the gripper to a pick-and-place machine. As these last two parts (B and C) do not present any microscopic features, they will be created with different manufacturing methods than the microscopic part (A).

## 3. Two-Scale 3D Printing

### 3.1. Microscopic Manufacturing

**Figure 2:**
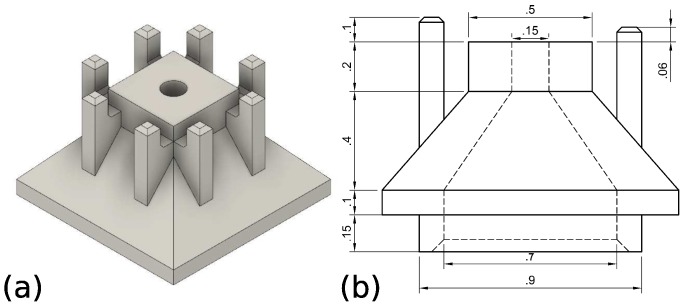
(**a**) Isometric view and (**b**) technical drawing of gripper head printed with Nanoscribe [13] (dimensions in mm).

As is apparent from Figure 1c, there are quite a few sub-millimetric slender features and intricate 3D shapes present in our design. As a result, a 3D printer capable of printing such features is deemed to be the best candidate. One of the most adequate 3D printing techniques capable of creating such a structure is two-photon polymerization, especially if we would want to shrink the components further in the future. In the present contribution, a Nanoscribe Photonics Professional GT was used to this end. The design shown in Figure 2 is printed in Nanoscribe IP-L 780 resist in dip-in configuration using a 25× objective.

### 3.2. Macroscopic Manufacturing

Now, as the microscopic gripper head is already printed in a photo-sensitive resist, it seems advantageous to make the macroscopic parts in the same (or a similar) material by using a stereolithographic 3D printer. In this way, we hope to achieve a perfect adhesion between both parts and avoid (thermal and/or mechanical) property mismatch. In addition, such 3D printers are also capable of intricate 3D designs with sub-millimetric features [14]. To this end, we have here used an Autodesk Ember 3D printer. For this printer, the UV-polymerisation is initiated by a digital projector resulting in a pixel-size of 50 μm. The resist used is Autodesk PR48 with a slicing distance of 25 μm.

**Figure 3:**
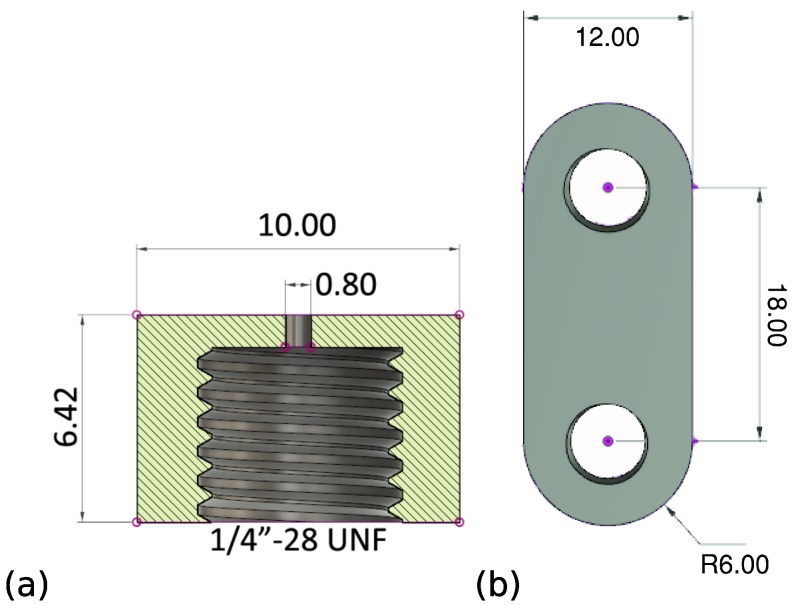
(**a**) Macroscopic connection part to liquid supply [13] (**b**) Macroscopic connection to stage [13] (dimensions in mm).

The design of the tip-holder is shown in Figure 3a. Note how we can print the 1/4” screw-thread directly into the component, allowing to connect the tubing. This tube is then connected to a CETONI Nemesys injection pump for the supply of water. Also note how the central 0.8 mm opening will be connected to the 0.7 mm opening in the microscopic component. In the microscopic part (Figure 2), this liquid channel is then further reduced in a funnel-design to a final opening of 0.15 mm.

In Figure 3b the supporting structure allowing to connect the gripper to a movement stage is shown. This piece will be clamped between the fitting and Figure 3a. On the other end, it will be similarly clamped between a screw and metal rod, which is itself connected to the movement stage. The final composition of all three components is shown in Figure 1a.

### 3.3. Bonding of Microscopic and Macroscopic Components

While the creation of the three pieces described above is quite trivial with the right equipment, the difficulty is in how to place the microscopic component correctly on top of the macroscopic component in Figure 3a and create a strong bonding. A bonding which should avoid blocking the small liquid channel.

**Figure 4:**
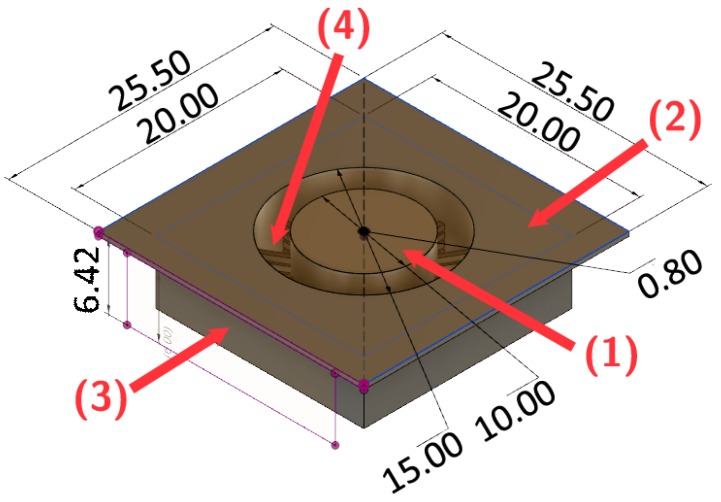
Tip holder design to conform to the Nanoscribe sample holder (dimensions in mm). (1) is the component shown in Figure 3a, (2) mimics the common glass substrates used in Dip-in configuration. (3) adds some rigidity as the small thickness of part (2) otherwise leads to a curved interface. (4) are a series of tiny bars connecting (1) to the holding structure [13].

In the current manuscript, the chosen manufacturing procedure was to write the microscopic component directly on top of the macroscopic component (i.e., the tip-holder shown in Figure 3a). As two-photon polymerization is capable of freely selecting the points that need to be polymerized, there is no difficulty in producing an open channel. Yet, in order to do this, we need to be able to insert the printed component into our Nanoscribe Photonics Professional GT. However, it only accepts standard-shaped glass slides or wafers as a target substrate on which to start writing. As such, we have had to design an envelope structure around our tip-holder, so that it conforms to a substrate size that the Nanoscribe sample holder can accept. Its design is shown in Figure 4. To summarize, we have mimicked a 1 inch square glass plate (indicated by (2) in the figure) surrounding the tip-holder (1). The tip-holder is held in place by a series of thin bars (4), which we can easily snap off after manufacturing. Note also that we have had to add extra thickness (3) to component (2) as the thin sheet started to deform after development.

Now, the final procedure to manufacture the combined component is as follows. The component in Figure 4 is printed with the Autodesk Ember. This component is then inserted into the sample holder of the Nanoscribe Photonics Professional GT. A drop of IP-L is placed on top of component (1) of Figure 4. The 25× objective is then allowed to dip into this drop to start writing the structure of Figure 2. Yet, before we can launch the printing, two more steps need to be performed first.

The first step is to find the liquid-solid interface, so that we can have a z-reference for the print. The in-build interface detection method by Nanoscribe is quite precise (±1 μm) but depends on the presence of a sufficiently large contrast in refractive index between the resist and the solid. The necessity of this is exemplified by the fact that even normal glass substrates need to be coated with ITO (Indium Tin Oxyde) to increase this contrast. Therefore it is quite inevitable that the refractive index contrast between the cured resist (Autodesk PR-48) and the liquid Nanoscribe IP-L 780 resist is not large enough to allow for an accurate automatic interface detection, due to the large similarity of the two materials. As adding an intermediate coating step could reduce the adhesion between both components, we chose to perform the interface finding manually based on the optical images of small residues on top of the printed part shown by Nanoscribe in direct imaging mode. As the obtained accuracy in this way is very poor and to account for any remaining non-flatness of the macroscopic component, we therefore added a 0.15 mm extension of the pyramid structure below the zero-height reference. As re-illumination of already polymerised voxels has no detrimental effects, this allows us to make sure that the pyramid is firmly attached to the tip-holder.

**Figure 5:**
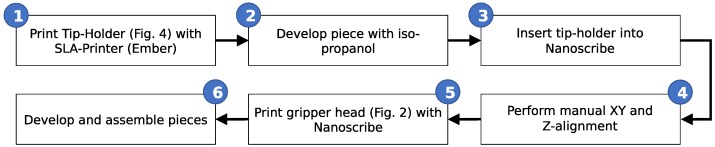
Flow chart for the manufacturing of the two-scale 3D printed capillary gripper.

A second reference that is still needed is the position of the centre of the liquid channel. This centering was also performed manually by noting the stage coordinates where the top, bottom, left and right edges of the channel are centered in the camera’s field of view. From this, the center point can be calculated. While the precision of this procedure could be as bad as a few tens of microns, this does not impact the final performance of our capillary gripper as the connecting channel has a sufficiently large diameter to be only mildly affected by such a misalignment of the two channels. The full workflow for this manufacturing approach is shown in Figure 5.

### 3.4. Manufacturing Results and Discussion

**Figure 6:**
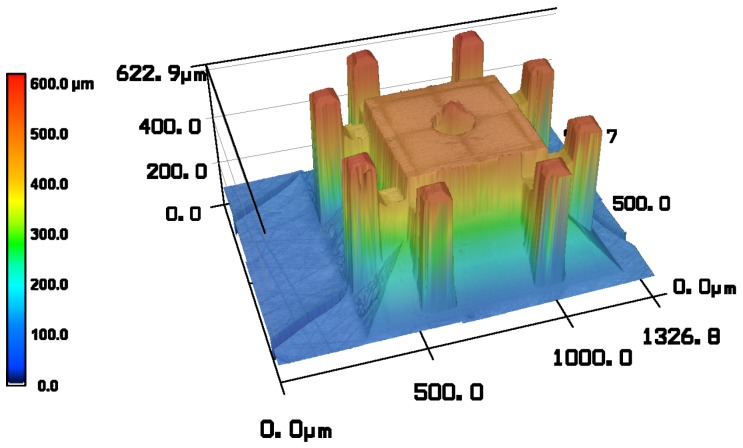
Metrology of the final picking head [13].

In Figure 6, the micrometric gripper head was measured by a confocal microscope (Keyence VK-X200, Keyence, Osaka, Japan). This clearly demonstrates that the chosen two-photon polymerisation manufacturing method is capable of faithfully reproducing the micrometric features of our design.

As will be shown in Section 5, the produced capillary grippers could pick and place the target components correctly. Nevertheless, there were a few manufacturing issues that appeared from time to time. First, we noticed that liquid leaks were sometimes present in the system. These could be due to limits in the manual alignment described above or could be due to cracks growing in the tip holder. Indeed, the resist employed by the Ember (PR-48) suffers of such cracking problems when continuously exposed to day light. A second issue that appeared from time to time was the occurrence of micro-explosions when polymerising with the Nanoscribe. Presumeably, a non-perfect cleaning step of the printed tip-holder could result in some debris floating in the IP-L. Illumination of this debris resulted in the appearance of explosions and bubbles, which resulted in a non-working gripper head. Although no extensive study was performed into these two issues, we do not consider either of them as an intrinsic problem of the proposed hybrid manufacturing method, but rather ‘solveable’ by optimisation studies (e.g., different resists for the Ember and lower illumination intensities for the Nanoscribe).

With respect to the total manufacturing time, we can note that the production of the tip holder (and its envelope) takes approximately 1.5 h to print with the Ember, whereas writing the gripper head with the Nanoscribe takes close to 5 h (including manual preparation tasks and development steps). As such, it is difficult to speed up the production process significantly in a cost-effective manner due to the prohibitive cost of buying additional Nanoscribe Photonics Professional GT units. On the other hand, the proposed two-scale 3D printing approach is optimally suited for complex 3D designs, both in the macroscopic components as in the microscopic components.

## 4. Metal+Moulding Manufacturing

### 4.1. Macroscopic Manufacturing

**Figure 7:**
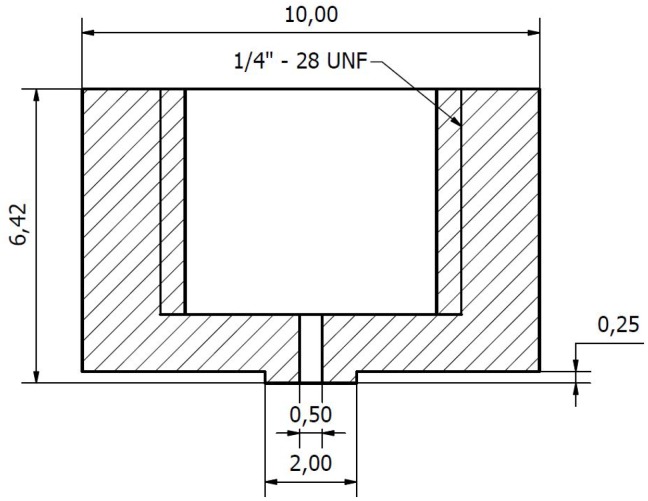
Updated design of the liquid connection device (dimensions in mm).

When looking at the designs in Figure 3, it is clear that none of these macroscopic components really requires the use of a 3D printer. Simple metal milling and drilling tools can create both components, which is what we have done for the second manufacturing approach. Due to reasons explained in the next subsection, a small modification of the tip-holder was necessary (an extra recess surrounding the microchannel) and the updated design is shown in Figure 7.

### 4.2. Microscopic Manufacturing and Bonding

The bottleneck in the previous hybrid manufacturing method was the production of the gripper head directly on top of each macroscopic component. The process that we will investigate now is how to use the technique of micromoulding to speed up the manufacturing. Clearly, a straightforward moulding of the complete gripper is not easy due to the complicated 3D design of the gripper with an internal channel. However, as the macroscopic components are easily manufactured using standard metal tooling, we really only need to focus here on the replication of a microscopic gripper head onto the metal tip holder.

The first concern here is how to preserve the microscopic details of our gripper head in the mould. Fortunately, this problem is already solved as replication with PDMS-moulds has already been shown in literature to faithfully reproduce micron-sized features created using two-photon polymerisation [15]. As such, we can use the same microscopic manufacturing method for the creation of the original gripper head, provided we simplify the design slightly so that moulding and demoulding can occur. This implies, for instance, that the funnel design of the microchannel in Figure 2 is replaced by a straight channel or an inverted funnel. The mould is obtained by casting a mixture of 10:1 PDMS/curing agent over the IP-L gripper in a vacuum environment to remove any bubbles. It is then cured over night at 65 °C and demoulded. Note that it is vital that the gripper undergoes a silanisation (1 h in a vacuum bell with an open reservoir of Hexamethyldisilazane) before the PDMS is poured, to allow for an easy detachment.

Another concern is how to combine the replicated gripper head with the tip-holder and ensure that there is a good adherence between the two components. To this end, replication and bonding is combined into a single step by applying the UV-illumination when the liquid resin is in contact both with the metal tip-holder as with the mould. Not only does this ensure a good bonding but it simplifies the total process significantly. Note that IP-L and Fun-to-do unpigmented standard blend were both tested successfully and never lead to liquid leakage, which qualitatively indicates that the bonding was indeed sufficiently strong.

**Figure 8:**
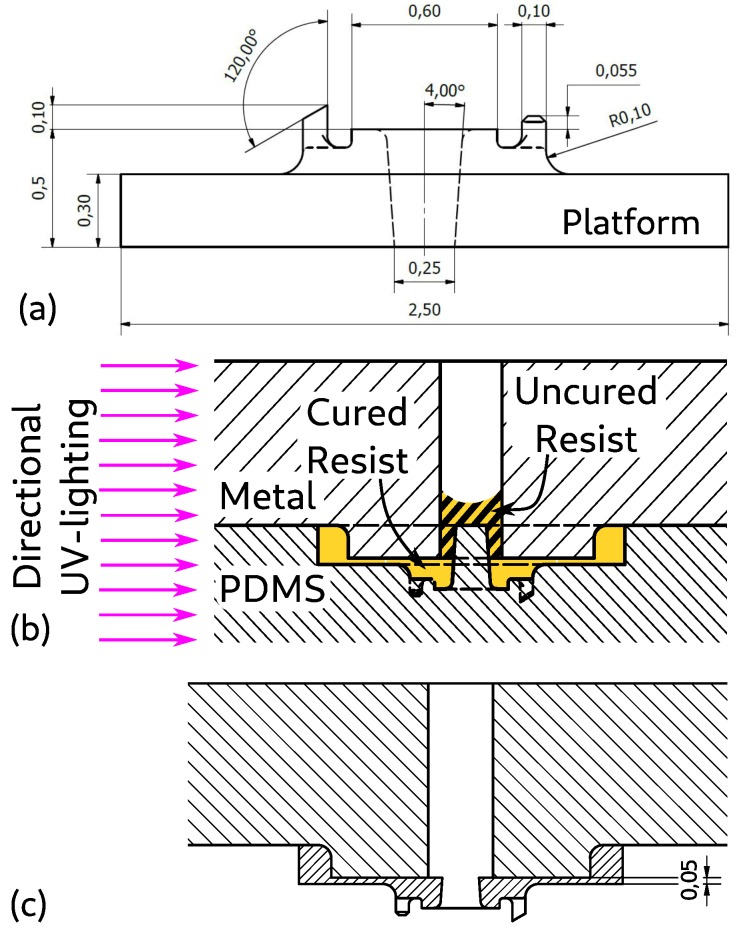
(**a**) Micromanufacturing design for Nanoscribe Manufacturing (dimensions in mm) (**b**) Zoom on the moulding composition near the liquid channel. (**c**) Zoom on the final gripper geometry.

The remaining issue is how to avoid obtaining a blocked microchannel. This will be achieved by a combination of design modifications and a special UV-illumination technique. The extra recess for the macroscopic component was already described. For the gripper, note how the head is now placed on a small platform in Figure 8a. The central hole for the microchannel goes through this platform and in this case even up to the substrate (see Figure 8a). Upon moulding, the platform becomes a recess and the hole becomes a pillar that sticks out above the level of the recess (Figure 8b). Now, when liquid resist is present in the mould and the metal piece is put in position, the pillar easily fits into the hole of the metal piece (see Figure 8b). However, if we would now polymerise all the resist, we would end up with a blocked channel as liquid resist will creep up into the micro-channel. This is avoided here by using directional UV-lighting that only comes from the side, not from the top or bottom (in practice we rotate the piece to illuminate it from all 4 sides). As the metal piece is non-transparent to UV-rays, the liquid resist inside the microchannel is not polymerised (region indicated by the hatched lines in Figure 8b). The final geometry of the gripper, upon retraction of the mould, is shown in Figure 8c.

**Figure 9:**
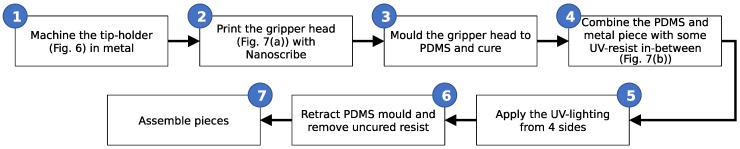
Flow chart for the manufacturing of the moulded gripper head on top of a macroscopic component.

Note that several other minor modifications were also applied to the design with respect to Figure 6, such as the presence of the releasing pillars only in a single direction and a modification of the releasing pillar shape in an attempt to optimise the performance of the gripper. The full workflow for this manufacturing approach is shown in Figure 9.

### 4.3. Manufacturing Results and Discussion

**Figure 10:**
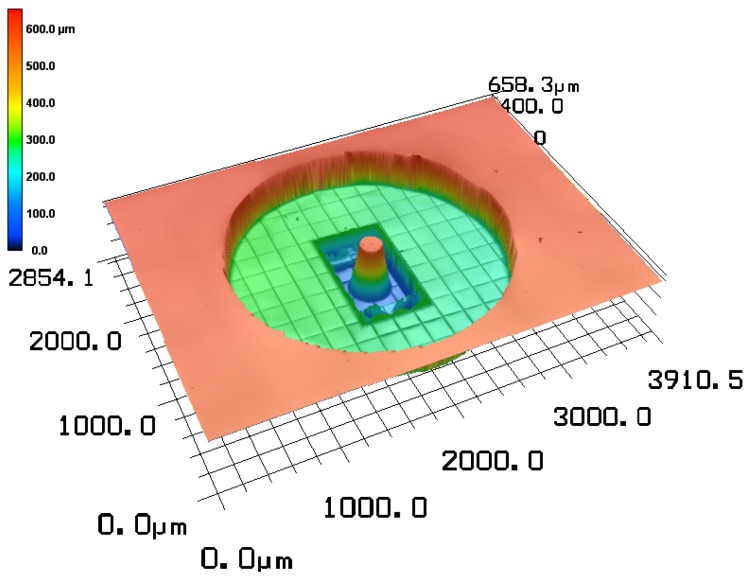
Metrology of the gripper head mould in polydimethylsiloxane (PDMS).

In Figure 10, the result of the confocal measurement of the mould is shown. As anticipated from literature [15], all of the sub-millimetric features are correctly preserved. More importantly, no sign of blocked bubbles are seen in the mould, even though there were many ‘dead-ends’ in the design. This is due to the casting in a vacuum chamber and the gas permeability of PDMS. In our limited testing, we could also easily make up to 7 assembled grippers from a single PDMS mould. In 2 of these cases, there was nevertheless a small membrane blocking the channel, presumeably due to spurious reflections from the UV-light. This blockage was however easily overcome by applying some pressure with the liquid syringe pump. After assembling 7 grippers, the mould was slightly damaged. It is expected that one could increase this number significantly by a careful deposition of a silane layer before each replication step or by the application of a more permanent chemical vapour deposited layer of Parylene-C [16]. By a similar deposition of a Parylene-C coating on the master, the amount of PDMS moulds that can be created per Nanoscribe master was shown to be arbitrarily large [17].

Now, with respect to the manufacturing time required to reach this result, the micro-manufacturing step takes more or less the same time than the previous design, i.e., 5 h. The moulding step in principle should only add about 3 h. However, in our experiments, we have chosen to leave the PDMS to cure overnight at 65 °C. In short, the initial tooling step takes about a day. However, once this is completed, creation of a replication head is just a matter of minutes and could easily be automated. As such, the current hybrid manufacturing method could be used in an industrial context and allows for the low-cost replication of microscopic gripper heads. To increase the productivity even further and parallellise the manufacturing, one can always create more high-precision moulds from the same master, as this process is non-destructive.

Another advantage of the present manufacturing approach is that we can easily use different UV-sensitive resists having different mechanical or other properties. For instance, the use of flexible UV-curable materials (such as UV-sensitive PDMS) could prove interesting.

Finally, we would like to mention that this moulding process could also be combined with 3D printing of the tip-holder as in the previous approach, without the need for the envelope structure. Indeed, resists are available that are opaque to UV-rays for the wall thicknesses considered here (>1 mm) [14]. As such, the directional UV-illumination will not penetrate into the micro-channel and it will remain unobstructed. This combination of manufacturing techniques (3D printing + moulding) would allow for more complex 3D designs in the macroscopic parts than in the microscopic part, but at a much faster total manufacturing speed.

## 5. Capillary Gripping

**Figure 11:**
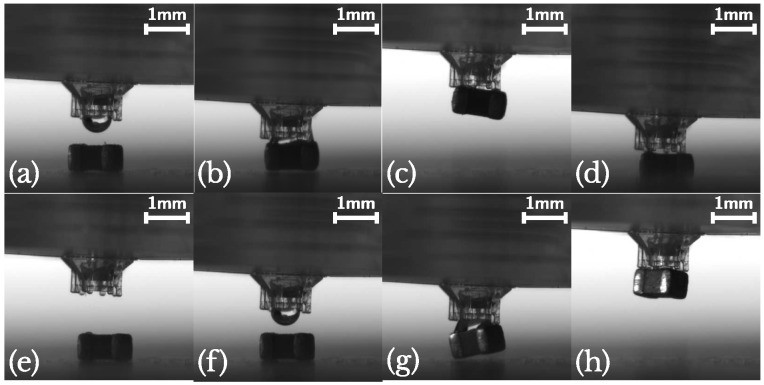
(**a**–**e**)—Pick and place sequence with the gripper from Section 3. (**e**–**h**) correct repicking of the component [13] (see video S1 in the Supplementary Materials).

Figure 11 shows the manipulation sequence with the gripper from Section 3: first, positioning (a) occurs: a droplet of 100 nL is sent to the picking surface and the gripper is aligned with the SMD component. Then the tool is moved downwards (b) and the component is picked up. To release the component, the working fluid is sucked back into the internal channel (c), the gripper is moved downwards again (d) and the component is released (e). As almost no liquid is left on the SMD component after the releasing operation, the re-picking performance is not influenced (Figure 11f–h). A video showing this sequence is available in the supporting information.

With the gripper from Section 4, a preliminary study was performed in order to investigate the positioning precision. In order to be independent of the precision of the used stage, no lateral displacements were performed with the stage, only vertical ones. The substrate was mounted on top of an inverted microscope (Leica microscope with a 2.5× objective). Using a glass substrate, this allowed a measurement of the absolute displacement of the centre of the component and the angular rotation from 13 subsequent pick and place actions. For the displacement, a systematic bias of 47 μm was found combined with a random standard deviation of 29 μm. For the rotation, we obtained 0.12 ± 5.5°.

**Figure 12:**
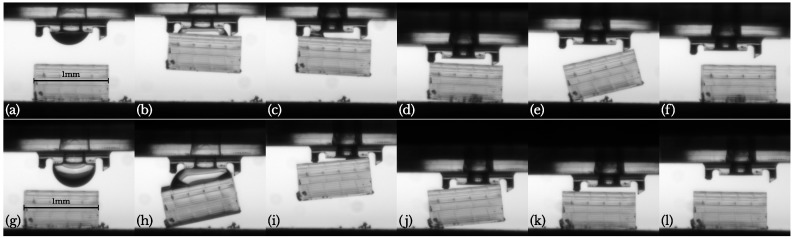
(**a**–**f**)—correct picking but faulty release sequence (**e**,**f**) with the gripper from Section 4 (see video S2 in the Supplementary Materials). (**g**–**l**)—correct *single-pillar* pick and release sequence (pillars on the right not in contact with the component) (see video S3 in the Supplementary Materials).

On more than one occasion however, the releasing procedure failed as is shown in Figure 12a–f. Although the component disconnected correctly from the droplet and the shorter pillar, the detachment from the longest pillar was not successful (e). The adherence to this pillar could even be large enough to lift the entire component. But eventually it disconnects and lands in an uncontrolled way (f). This was observed with the flat pillars from Section 3 and with the new slanted pillar from Section 4. A possible solution that could be investigated in the future is to increase the redrawal acceleration to overcome this residual adherence.

An interesting possibility to improve our gripper design, can be seen in Figure 12g–l. Here, only the shortest pillar is used and the component is aligned with 3 edges of the gripper surface. This leads to a larger inclination and detaching moment for the gripper upon touching the substrate. Due to this, the component disconnects from the gripper surface at a larger height (j) and inertia allows it to disconnect from the pillar as well, leading to a clean separation (k–l). The mechanism could be understood better from the video in the Supplementary Materials.

Another advantage of only using a single pillar, is in the time scale needed to release the component. Not yet mentioned, is the fact that not all liquid can be retracted in the tube. As can be clearly seen in Figure 12c, a small liquid bridge remains on the left of the channel. For releasing the component, this should be allowed to evaporate (≈40 s for water) (d). In comparison, the quantity of liquid after retraction shown in Figure 12i is clearly much smaller, allowing for a faster placing.

## 6. Conclusions

In the present paper, we have experimentally validated a design for a capillary gripper for submillimetric components. The gripper can be connected to tubing for liquid supply and withdrawal. The release mechanism is completely passive and based on asymmetric pillars with a placement precision of ±29 μm (for a 1 × 0.5 (mm) component).

A major focus of the paper was on the development of hybrid multi-scale manufacturing methods, capable of integrating micron-sized 3D features into a centimetric component. A first development focussed on ultimate 3D complexity of both the ‘macroscopic‘ component as the ‘microscopic’ parts by using two 3D printers; an Autodesk Ember (Stereolithographic 3D printer) and a Nanoscribe Photonics Professional GT (two-photon polymerisation) respectively. By printing the gripper head directly on top of the macroscopic part, a good adherence was assured. However, this creates an inherently slow manufacturing process with a maximum of 1 or two pieces produced per day.

The second manufacturing procedure introduced, consisted in replicating the microscopic gripper head directly from a PDMS mould onto a metallic part. Assuming that the metal parts can be produced straightforwardly, this method could produce tens of completed grippers in a day. This is of course at the expense of a higher start-up cost in which an appropriate master and its PDMS-mould needs to be manufactured (≈1 day). The production of a non-obstructed micro-channel without additional post-processing steps needed an adapted design and use of directional UV-lighting to avoid polymerisation inside the channel, which was successfully demonstrated.

## Supplementary Materials

The following videos are available online at https://www.mdpi.com/2072-666X/10/4/224/s1. Video S1: PickPlace_EmberNanoscribe.mkv, Video S2: PickPlace_BadRelease.mkv, Video S3: PickPlace_SinglePillar.mkv.
